# Protective Strategies Against Glyphosate and Glyphosate-Based Herbicide Toxicity: Mechanisms, Experimental Evidence, and Translational Limitations

**DOI:** 10.3390/nu18101573

**Published:** 2026-05-15

**Authors:** Kaja Hanna Karakuła, Ryszard Sitarz, Alicja Forma, Dominika Przygodzka, Grzegorz Teresiński, Dariusz Juchnowicz, Grzegorz Buszewicz, Jacek Baj

**Affiliations:** 1 Department of Clinical Neuropsychiatry, Medical University of Lublin, 1 Głuska St., 20-439 Lublin, Poland; 2 Department of Forensic Medicine, Medical University of Lublin, 8b Jaczewskiego St., 20-090 Lublin, Poland; formaalicja@gmail.com (A.F.); dominika.przygodzka@umlub.edu.pl (D.P.); grzegorz.teresinski@umlub.edu.pl (G.T.);; 3 I Department of Psychiatry, Psychotherapy and Early Intervention, Medical University of Lublin, 1 Głuska St., 20-439 Lublin, Poland; ryszard.sitarz@umlub.edu.pl; 4 Department of Psychiatry and Psychiatric Nursing, Medical University of Lublin, 7 Chodźki St., 20-093 Lublin, Poland; dariusz.juchnowicz@umlub.edu.pl; 5 Department of Correct, Clinical, and Imaging Anatomy, Medical University of Lublin, 4 Jaczewskiego St., 20-090 Lublin, Poland; jacek.baj@umlub.edu.pl

**Keywords:** glyphosate, glyphosate-based herbicides, oxidative stress, inflammation, mitochondrial dysfunction, experimental toxicology

## Abstract

(1) Background: Glyphosate (GLY) and glyphosate-based herbicides (GBHs) are widely used agrochemicals. Experimental studies have reported oxidative stress, inflammatory activation, mitochondrial impairment, endocrine-related effects, and organ injury following GLY/GBH exposure; however, candidate mitigation approaches have not been comprehensively summarized across experimental systems. (2) Methods: This structured narrative review followed SANRA recommendations. PubMed, Scopus, Web of Science, and Embase were searched (January 2004–January 2026). In total, 37 experimental studies met the inclusion criteria, describing 23 compounds categorized as vitamins, antioxidants, or enzyme modulators, dietary supplements, plant extracts, humic substances, hormonal modulators, and other natural compounds. (3) Results: Across models, reported protective effects most consistently involved attenuation of oxidative damage, including reductions in lipid peroxidation, oxidative DNA damage markers, and partial restoration of endogenous antioxidant defenses. Several interventions also modulated inflammatory signaling, apoptosis-associated markers, and stress response signaling. Protective effects were generally dose-dependent and more frequently observed in pre-treatment or co-exposure paradigms; complete normalization of outcomes was uncommon. Interpretation across studies was limited by heterogeneity in exposure conditions, test systems, endpoints, and, critically, by differences between pure GLY and GBHs. (4) Conclusions: Experimental evidence supports the mechanistic plausibility of antioxidant and stress response modulation as candidate approaches to mitigate GLY/GBH-induced toxicity. However, substantial methodological variability, frequent use of high-dose or non-representative exposure paradigms, and the absence of human interventional data limit translational relevance. Future studies should prioritize standardized, formulation-specific designs with exposure scenarios aligned to real-world conditions and include systematic safety assessment of proposed interventions.

## 1. Introduction

Glyphosate (GLY), the most widely used herbicide globally, was introduced in 1974. Glyphosate-based herbicides (GBHs) are pesticide formulas that contain GLY as an active ingredient. Use in agriculture has a wide range, from presowing to harvest, and it has also found use in parks and home gardens [1]. GLY is resistant to complete degradation and can persist in the environment for extended periods. In soil, its breakdown depends largely on microbial activity and temperature, pH, and soil composition [2,3]. GLY residues are found in drinking water, agricultural soils, animal feed, groundwater, rain, and even air [4]. Researchers have shown that it can also be found in plant-derived commodities such as cereals, legumes, canola, and soya [5,6,7].

The International Agency for Research on Cancer classified GLY as a ‘probable human carcinogen’ in 2015 [8]. However, in the same year, the European Food Safety Authority and in 2017, the US Environmental Protection Agency concluded that ‘glyphosate is unlikely to pose a carcinogenic risk to humans’ [9,10]. An increasing number of experimental and observational studies have reported associations between GLY exposure and processes implicated in tumorigenesis, reproductive disturbances, low-grade inflammation, and oxidative stress [11]. Moreover, GBHs are found to be more toxic than GLY alone due to the presence of additional adjuvants in the formulation, such as surfactants, which enhance cellular uptake and may promote toxic effects themselves [12]. Centering solely on GLY can potentially underestimate real-world health risks [13].

Much of the literature has focused on oxidative stress as a central mechanism of toxicity (OS) [14]. OS refers to the process in which an excess of reactive oxygen species (ROS) is generated, resulting in damage to proteins, lipids, cell membranes, and, most critically, genetic material [15]. OS is well known to contribute to the development of diabetes, neurodegenerative diseases, carcinogenesis, liver toxicity, and other conditions [16,17]. Experimental models have reported disruption of the pro- and antioxidant balance by inducing OS [18].

GBHs dysregulate the oxidant balance mainly by increasing oxidative stress markers, e.g., lipid peroxidation (LPO) and malondialdehyde (MDA) levels, and depletion in antioxidant enzymes, e.g., superoxide dismutase (SOD), catalase (CAT), and glutathione peroxidase (GPx) [19,20]. Additionally, GLY disrupts mitochondrial function by impairing complex I of the mitochondrial respiratory chain and deregulating iron transport-related genes, leading to redox imbalance. At environmental concentrations, GLY may induce the expression of cytochrome P450 enzymes involved in biotransformation, alter the regulation of ATP-binding cassette transporters, and inhibit the redox-sensitive nuclear factor erythroid 2-related factor 2 (Nrf2) signaling pathway, further exacerbating OS and cellular dysfunction [21].

Furthermore, GLY can disrupt metabolic pathways and endocrine systems [22]. It was observed to cause functional and histopathological changes [23]. In the liver, GLY exposure has been associated with hepatocyte degeneration, steatosis, and fibrosis [24,25,26]. In the kidneys, tubular necrosis, glomerular atrophy, and interstitial inflammation are common, leading to reduced filtration efficiency [27]. Nevertheless, not all studies observed the same effects of GLY-toxicity [28].

It is important to note that a substantial part of the literature reporting on GLY and GBHs’ toxicity is derived from in vitro models and small-sample animal in vivo experiments [14,29]. They are designed primarily to explore mechanistic pathways such as oxidative stress, inflammatory signaling, mitochondrial dysfunction, endocrine perturbation, and apoptosis [14,29,30]. These studies are valuable for identifying biological plausibility and hazard-related mechanisms, but they should not be interpreted as direct evidence of human health risk without careful consideration of dose level, formulation, route of administration, and exposure relevance [31].

In response to these findings regarding the toxic effects of GLY and GBHs, increasing attention has been directed towards interventions with potential protective compounds that may mitigate their toxicity. Vitamin C and E [32], *N*-acetylcysteine [33], melatonin [34], quercetin [35], and other plant-derived bioactive compounds [36] are among the most frequently investigated agents. Researchers most often focus on their ability to attenuate oxidative damage, support endogenous antioxidant defenses, modulate inflammatory signaling, and reduce apoptosis or tissue injury. However, interpretation of these findings requires caution due to the available studies that differ substantially with respect to herbicide formulation, administered dose, route and duration of exposure, timing of intervention, and biological model. This methodological heterogeneity complicates direct comparison across studies and limits the strength of translational inferences.

The article aimed to examine and summarize the available evidence on the potential protective effects of various vitamins, antioxidants, natural extracts, and other compounds against the harmful effects of GLY and GBHs. By analyzing evidence from existing research, prospective strategies to mitigate the health effects of GLY toxicity could emerge.

## 2. Materials and Methods

This structured narrative review followed SANRA recommendations [37] and incorporated a structured literature search across major biomedical databases. The review covered studies published between January 2004 and January 2026. English-language publications were identified by structured searches of PubMed, Scopus, Web of Science, and Embase. The search strategy included the following keywords: (glyphosate OR GLY OR glyphosate-based herbicides OR GBHs) AND (mitigat* OR alleviat*).

**Figure 1:**
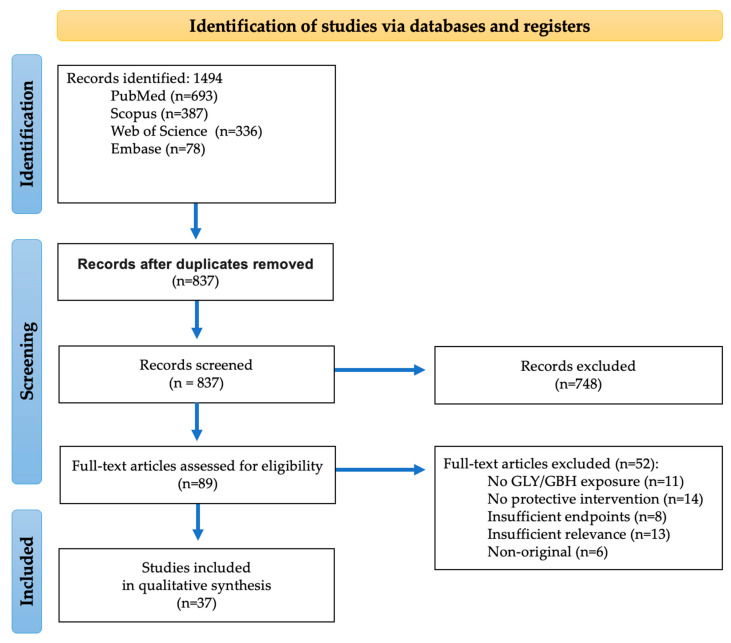
Study selection flow diagram.

A total of 37 peer-reviewed original research articles met the predefined inclusion criteria. The study selection process is summarized in Figure 1.

### 2.1. Study Selection

#### 2.1.1. Inclusion Criteria

The following inclusion criteria were applied: (1) experimental studies in animals or humans evaluating the toxicity of GLY or GBHs; (2) studies reporting mitigating effects of interventions against GLY- or GBH-induced toxicity; (3) reporting biochemical, molecular, or morphological endpoints relevant to GLY/GBHs toxicodynamics; and (4) in vivo or in vitro studies.

#### 2.1.2. Exclusion Criteria

The articles that met any of these criteria were excluded from the analysis: (1) non-original publications; (2) studies deemed insufficiently relevant to the objectives of this review; (3) works focused exclusively on other pesticides or toxicants without a clear GLY/GBHs component; (4) lack of full text.

#### 2.1.3. Selection Process

Data were extracted independently by two reviewers using a standardized form, including details on study type, exposure conditions (GLY or GBHs), model (in vivo or in vitro), dose, duration, and measured outcomes. Titles/abstracts were screened, full texts were assessed for eligibility, and duplicates were removed.

Study relevance assessment (0–2 scale). Relevance was assessed independently by two researchers (KHK, RS) on a three-point scale: 2—the study fully addressed at least one of the main objectives of this review; 1—the study partially addresses the objectives; 0—the study does not address the objectives. Differences in scoring were resolved by discussion and consensus.

### 2.2. Data Extraction

The collected data were grouped according to the mitigating substance used. The included studies employed in vitro and in vivo models to evaluate biochemical markers of oxidative stress and histopathological alterations associated with GLY or GBH exposure. The analysis was narrative, emphasizing biochemical results and histology/function.

Given considerable heterogeneity in model systems, exposure regimens, formulations, and outcome measures, a meta-analysis was not feasible. The findings were therefore synthesized narratively.

## 3. Results

**Figure 2:**
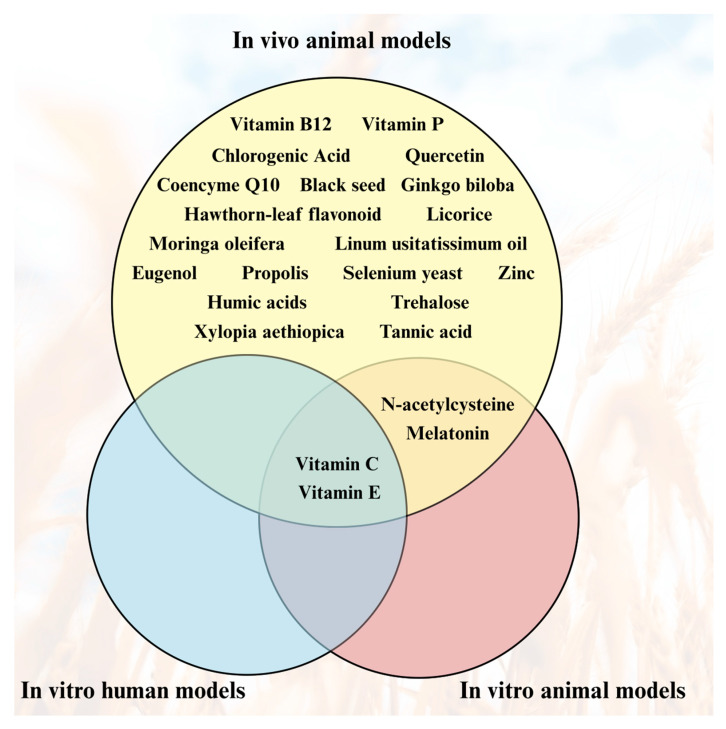
Experimental systems distribution: substances tested against glyphosate toxicity across three experimental systems—in vivo animal models (top), in vitro human cell models (bottom-left), and in vitro animal cell models (bottom-right); overlaps indicate substances evaluated in multiple systems.

A total of 37 studies met the inclusion criteria and were included in the qualitative synthesis. As demonstrated in Figure 2, nearly all of the included substances were exclusively evaluated in in vivo animal models. Only *N*-acetylcysteine and melatonin were additionally assessed in animal in vitro systems, whereas vitamins C and E were the only compounds evaluated across all three experimental models: animal in vivo, animal in vitro, and human in vitro models.

All the substances reviewed were categorized into the following groups: (i) vitamins [32,38,39,40,41,42,43,44], (ii) antioxidants and enzyme modulators [33,35,45,46,47,48,49,50], (iii) dietary supplements and natural extracts [27,36,51,52,53,54,55,56,57,58,59,60,61], (iv) humic substances [62,63], (v) hormonal modulators [34,64,65], and (vi) other natural compounds [66,67,68]. Detailed findings are summarized in Table 1.

Across the studies, modulation of oxidative stress was the most consistently reported protective mechanism [27,32,33,35,38,39,42,43,44,47,48,49,51,54,55,56,57,58,64,66,67,68]. However, the significance of the effect varied substantially between compounds and models. This was most often reflected by reduced lipid peroxidation (e.g., lower MDA levels) [32,33,35,42,43,44,47,48,49,51,54,56,57,58,66,67,68] together with improved antioxidant defenses (SOD, CAT, GPx, GSH) [38,52,53,55,58,66,67,68]. Anti-inflammatory effects were also common. The most commonly reported anti-inflammatory effects were decreases in tumor necrosis factor alpha (TNF-α) and nuclear factor kappa B (NF-κB) activity [45,46,52,53,58,68], with partial restoration of interleukin 10 (IL-10) in several models [38,58]. In some experimental settings, improvements in oxidative and inflammatory markers were accompanied by reduced activity of apoptosis-related pathways, such as the Bcl-2-associated X protein (Bax)/B-cell lymphoma 2 (Bcl-2) balance and caspase-3 [40,41,43,45,46,48,50,53,55,64,68].

The protective effects showed a tendency to be dose-dependent and were often more evident in experimental settings involving either pre-treatment with the protective compound or its co-administration with GLY/GBHs. Nevertheless, complete normalization to control group levels was rarely achieved. In several studies, interpretation of protective effects was further limited by small sample sizes, short exposure periods, and limited long-term follow-up.

**Figure 3:**
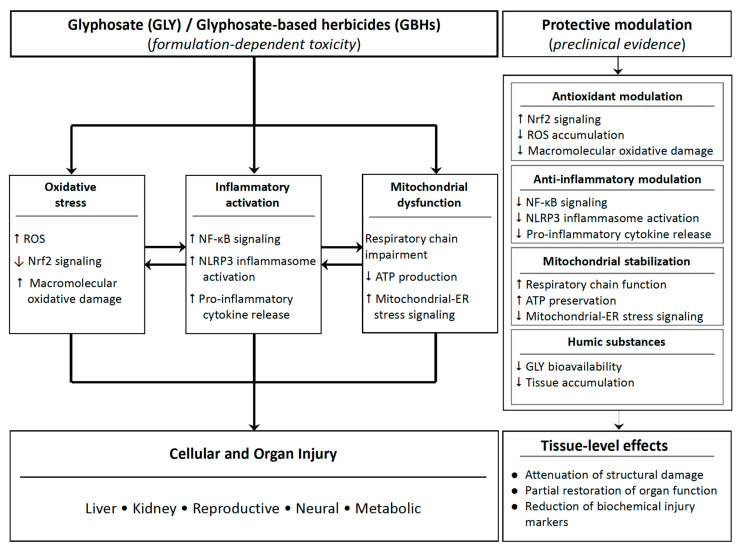
Conceptual synthesis of shared mechanisms of glyphosate/GBH-induced toxicity and their modulation by protective compounds.

To integrate the shared mechanistic pathways identified across the included studies, a conceptual model summarizing GLY/GBH-induced toxicity and proposed protective modulation is presented in Figure 3.

### 3.1. Vitamins

#### 3.1.1. Vitamin B12

Ngatuni et al. [38] investigated the potential role of vitamin B12 (vitB12) in mitigating GBH-induced toxicity in a murine model, with particular attention to the timing and mode of administration. In one experimental group, vitB12 was orally co-administered with GBHs throughout the exposure period, whereas in another group, vitB12 was administered alone for the first 7 days, followed by combined treatment with GBHs for the remaining 56 days. The type of GBH was not specified [38].

GBH exposure resulted in a significant reduction in body weight compared to controls, and vitB12 supplementation did not reverse this effect. Most hematological parameters were decreased in the GBH-exposed group, except for lymphocyte counts, mean platelet volume, and platelet distribution width, which remained unchanged. Vitamin B12 stabilized several blood morphology parameters during GBH exposure.

GBH administration increased serum bilirubin, alanine aminotransferase (ALT), and aspartate aminotransferase (AST) levels. Co-supplementation with vitB12 normalized bilirubin levels but did not fully restore transaminase activity. An increased triglyceride-to-high-density lipoprotein (HDL) ratio was also observed following GBH exposure; vitB12 stabilized HDL levels but did not normalize the overall lipid profile.

Oxidative stress was assessed via glutathione (GSH) levels. GBH exposure decreased hepatic GSH and increased GSH levels in the kidneys, lungs, and brain. Vitamin B12 supplementation restored hepatic GSH and normalized GSH levels in extrahepatic tissues during co-exposure.

Additionally, GBHs elevated tumor necrosis factor-alpha (TNF-α) and reduced IL-10 levels, both of which were restored following vitB12 supplementation. Interferon-gamma (IFN-γ) levels were not significantly affected by either GBHs or vitB12 [38].

Taken together, the findings suggest that vitamin B12 may attenuate selected biochemical, oxidative, and inflammatory disturbances induced by GBHs, but its protective effects appear to be incomplete.

#### 3.1.2. Vitamin C and E

Seven studies evaluated vitamin C (vitC) and/or vitamin E (vitE) as potential protective agents against GLY or GBH-induced toxicity.

An early in vitro study (2004) assessed the cytotoxicity of GLY and its commercial formulation, Roundup 3 plus^®^ (concentrations: 10, 12.5, 15, 17.5, 20, 22, and 25 mM) in human HaCaT keratinocytes, examining the cytoprotective effects of vitC and vitE administered as pre- or co-treatment (concentrations: 100 and 200 µM). Cytotoxicity was measured using a colorimetric assay based on formazan formation, allowing determination of the concentration that reduces cell viability by 50%. Both vitamins demonstrated comparable radical-scavenging activity during co-exposure. Roundup 3 plus^®^ exhibited greater cytotoxicity than pure GLY, likely due to the presence of polyethoxylated tallow amine and other formulation components. Antioxidant concentration and incubation time appeared more critical for protection than antioxidant type. No additional benefit was observed with pre-incubation or combined vitamin administration. Optimal cytoprotection was achieved with a 75% vitC and 25% vitE mixture at 190 µM [39].

In a subsequent study, vitE (100 µM) showed protective effects against GLY- and Roundup-induced cytotoxicity (10, 12.5, 15, 17.5, 20, 22, and 25 mM), whereas vitC (100 µM) was effective primarily against Roundup^®^. Both vitamins reduced oxidative damage, increased the activities of antioxidant enzymes (glutathione reductase (GSSG-Red), GPx, SOD), and lowered lipid peroxidation in Roundup-treated cells. Despite enhanced GSSG-Red activity, intracellular GSH levels remained reduced. VitC decreased thiobarbituric acid reactive substances, while vitE increased them. Additionally, vitC enhanced CAT activity, whereas vitE reduced it [32].

In caprine ovarian granulosa cells, co-administration of vitC or vitE (50 and 100 µM) attenuated Roundup-induced (0.1, 2.0, and 4.0 mg/mL) oxidative stress and apoptosis, reducing nuclear fragmentation, chromatin condensation, and membrane damage. Antioxidant enzyme activities (SOD, CAT, GST) improved but did not reach control levels. VitC demonstrated greater efficacy than vitE. These findings were replicated in a later study [40,41].

Fréville et al. evaluated vitE (1 mg/L) in primary chicken granulosa cells exposed to increasing GBH concentrations (Gallup Super 360: 0.036, 0.36, 3.6 and 36 mg GLY eq/L). While GBHs did not induce overt cytotoxicity, vitE mitigated oxidative stress by decreasing total oxidant status and oxidative stress index and increasing total antioxidant status. Vitamin E partially restored progesterone production at selected doses, although key steroidogenic gene expression (steroidogenic acute regulatory protein (StAR), cytochrome P450 family 11 subfamily A member 1, 3β-hydroxysteroid dehydrogenase) remained unaffected [42].

In vivo, vitE supplementation (100 mg/kg/day via intraperitoneal injection) in female BALB/c mice attenuated GLY-induced (250 or 500 mg/kg/day via gavage) weight loss and oxidative damage, significantly reducing MDA levels and increasing GSH and CAT activity. GLY exposure decreased reproductive hormone levels, whereas vitE restored progesterone concentrations and increased estrogen levels. Furthermore, vitE mitigated GLY-induced upregulation of pro-apoptotic genes (Bax, caspase-3, caspase-9), downregulation of Bcl-2, and suppression of steroidogenesis-related genes (StAR, 3β-HSD) [43].

Overall, vitamins C and E demonstrated antioxidant and partial anti-apoptotic effects across in vitro and in vivo models, although their efficacy varied depending on formulation, dose, and experimental context.

#### 3.1.3. Vitamin P (Hesperidin)

One study evaluated vitamin P (hesperidin, HES) as a protective agent against GLY-induced testicular toxicity in Wistar rats. HES, a citrus-derived flavonoid, is known for its hypolipidemic, anti-inflammatory, anticancer, and antioxidant properties. Rats were exposed for 56 days to HES (100 mg/kg BW/day), GBH (Knockdown 48 SL, 787.85 mg/kg BW/day), or a combination of both via gavage. The GBH dose used in this study is much higher than the European acceptable daily intake for glyphosate. Therefore, these findings should be interpreted as reflecting a high-dose experimental toxicity model rather than typical dietary exposure.

GBH exposure impaired sperm parameters, whereas HES supplementation restored sperm motility to near-control levels and reduced the incidence of sperm abnormalities. HES also attenuated oxidative stress, as reflected by reduced MDA and total oxidant status (TOS), together with increased glutathione (GSH) and total antioxidant status (TAS), resulting in a lower oxidative stress index (OSI). Additionally, HES significantly reduced GBH-induced DNA fragmentation.

Histopathological examination revealed that GBH exposure caused degeneration of the germinal epithelium, vacuolization, seminiferous tubule atrophy, congestion of interstitial blood vessels, and the presence of immature germinal cells in the tubular lumen. Co-treatment with HES markedly improved testicular architecture, approaching that observed in control animals. No significant differences in testosterone levels were detected among the groups [44].

Collectively, hesperidin exerted antioxidant and histoprotective effects against GBH-induced testicular damage, although endocrine parameters remained unchanged.

### 3.2. Antioxidants and Enzyme Modulators

#### 3.2.1. Chlorogenic Acid

Chlorogenic acid (CGA), a compound with established antioxidant and anti-inflammatory properties, was evaluated in male Wistar rats exposed to GBHs (Knock-out^®^, 800 mg/kg BW). CGA was administered as a pre-treatment at doses of 12.5, 25, or 50 mg/kg BW for 49 days by gavage.

CGA dose-dependently attenuated GBH-induced oxidative stress, reversing elevated MDA levels and restoring reduced GSH, SOD, and CAT activities in blood as well as in brain, heart, liver, and kidney tissues. The highest CGA dose nearly normalized antioxidant enzyme activity and overall oxidative status across examined organs.

Similar dose-dependent improvements were observed in biochemical markers of hepatotoxicity and nephrotoxicity (AST, ALT, ALP, urea, and creatinine). Pre-treatment also reduced levels of 8-hydroxy-2′-deoxyguanosine, a marker of oxidative DNA damage, although values did not fully return to control levels.

Histopathological examination revealed GBH-induced lesions in the brain, heart, liver, and kidneys, which were mitigated by CGA in a dose-dependent manner. Notably, CGA administered alone did not produce adverse effects in any assessed parameter [33].

Overall, CGA demonstrated broad antioxidant and organ-protective effects against GBH-induced toxicity, with efficacy increasing in a dose-dependent manner.

#### 3.2.2. Coenzyme Q10

Coenzyme Q10 (CoQ10) is a mitochondrial antioxidant with documented anti-inflammatory and anti-apoptotic activity. Mutluay et al. (2025) [45] evaluated the protective effects of CoQ10 against GBHs-induced testicular and sperm toxicity in an adult male ICR mouse model. The study lasted one spermatogenic cycle, corresponding to 35 days. Mice received oral GBH exposure (Roundup^®^; 500 mg/kg BW/day) with or without CoQ10 co-treatment (200 mg/kg BW/day) for 35 days via gavage. GBH exposure caused testicular and sperm toxicity, including reduced testicular weight, histopathological disruption of seminiferous tubules, impaired sperm concentration, motility, viability, morphology, and membrane integrity, increased mitochondrial ROS, and hormonal disturbances (including reduced testosterone and increased luteinizing hormone). These changes were accompanied by increased OS—elevation of MDA, TOS, and OSI and reduction in GSH and TAS.

At the molecular level, GBH suppressed antioxidant signaling (Nrf2/Kelch-like ECH-associated protein 1 (Keap1)/heme oxygenase 1 (HO-1)), activated inflammatory signaling (toll-like receptor 4/NF-κB), and promoted apoptosis (increased Bax, decreased Bcl-2). CoQ10 co-treatment significantly reduced these alterations. Co-treatment significantly ameliorated the effects of GBHs on oxidative balance, reproductive hormone levels, testicular and sperm function, and apoptosis signaling [45].

These results indicate that CoQ10 has multi-target protective effects against GBH-induced male reproductive toxicity by attenuating oxidative stress, inflammation, and apoptosis. Nevertheless, the evidence remains preclinical, and a single high-dose exposure design limits translation.

#### 3.2.3. Eugenol

Eugenol (EUG) is a natural phenolic antioxidant evaluated in one study for its potential to mitigate glyphosate-induced testicular injury in an adult male rat model. Rats were orally exposed to 150mg/kg/day GBHs (Roundup^®^) over a 7-day period via gavage. Researchers observed toxicity characterized by histopathological damage, OS (increased MDA with depletion of GSH and antioxidant enzymes including SOD, CAT, and GPx), and broad activation of stress-related molecular pathways. The study assessed multiple signaling pathways related to cellular stress and injury, including PI3K/AKT signaling, endoplasmic reticulum stress, RAGE/NLRP3-associated responses, inflammatory mediators (e.g., NF-κB, TNF-α), apoptosis-related proteins (Bax, Bcl-2, caspase-3), and antioxidant regulators (Keap1/Nrf2).

Co-treatment with EUG at 100 mg/kg was associated with reduced oxidative stress, ER stress, and RAGE/NLRP3 inflammasome signaling, as well as with modulation of the PI3K/AKT pathway. These effects were accompanied by partial restoration of antioxidant enzyme activity and improvement of testicular histopathology [46].

In summary, EUG appears to act as a multi-target protective compound against GBH-induced testicular injury by modulating oxidative, inflammatory, ER stress, and apoptotic pathways based on short-term animal data.

#### 3.2.4. *N*-Acetylocysteine

*N*-acetylcysteine (NAC) was evaluated in three studies as a potential protective agent against GLY/GBH-induced toxicity. In a rat model, co-administration of NAC (160 mg/kg BW/day, via gastric gavage) significantly reduced MDA levels and restored GSH concentrations in blood as well as in brain, liver, kidney, and heart tissues. GBHs (Knockdown 48SL, GLY ~ 375 mg/kg BW/day, via gastric gavage) decreased SOD and CAT activities selectively in erythrocytes, likely showing their increased susceptibility to oxidative damage; NAC supplementation restored both enzyme activities to near-control levels. Improvements were also observed in biochemical markers of organ function, including reductions in serum AST, ALT, ALP, total protein (TP), creatinine, and urea, as well as normalization of cardiac injury markers (CK-MB, cTn-I). Histopathological damage—such as neuronal degeneration, hepatic sinusoidal dilatation, cardiac muscle degeneration, renal tubular necrosis, and glomerular alterations—was attenuated following NAC treatment [47].

In a subsequent study, NAC co-supplementation (NAC 160 mg/kg BW/day, via gavage; Roundup GLY ~ 375 mg/kg BW/day, via gavage) in Wistar rats reduced MDA levels (−71.8%), increased total antioxidant capacity (+75.4%), and upregulated NRF2 mRNA expression (+43%), supporting its role in oxidative stress modulation. Although AST and ALT levels decreased, they did not fully return to control values. NAC also reduced GBH-induced upregulation of pro-apoptotic genes (Bax, c-Myc) and partially attenuated histological liver damage. Increased expression of caspase-3 and PCNA observed in GLY-exposed animals was mitigated, although not completely normalized [48].

In caprine testicular cells, co-administration of NAC attenuated GBH-induced (NAC 100 µM; Roundup^®^ 10, 12.5, 15, 17.5, 20, 22, and 25 mM) apoptotic features, including nuclear pyknosis, tubular degeneration, and vacuolization. NAC reduced lipid peroxidation and restored antioxidant enzyme activities (SOD, CAT, GST) in a dose-dependent manner [49].

Collectively, NAC demonstrated consistent antioxidant and partial anti-apoptotic effects across multiple organ systems, although complete normalization of GBH-induced damage was not consistently achieved.

#### 3.2.5. Quercetin

Quercetin (QE), a flavonoid with antioxidant, anti-inflammatory, and anticancer properties, was evaluated by Soudani et al. [35] for its protective effects against GLY-induced oxidative stress and hepatotoxicity in rats. QE (20 mg/kg BW/day) was administered daily by gavage, whereas GLY (50 mg/kg BW) was injected intraperitoneally every two days.

GLY exposure significantly increased oxidative stress markers, including malondialdehyde (MDA; +69%) and hydrogen peroxide (+44%). QE supplementation reduced these elevations by approximately 23%, although values did not return to control levels. Similar partial improvements were observed for advanced oxidation protein products, protein carbonyls, and liver injury markers (AST, ALT, ALP, gamma-glutamyl transferase (γ-GT)), as well as albumin levels.

GLY induced DNA damage in hepatic tissue, evidenced by DNA smearing on agarose gel electrophoresis, and increased total metallothionein (MT) levels (+70%) together with upregulation of MT-I and MT-II gene expression. QE attenuated DNA fragmentation and reduced MT levels, concomitantly downregulating MT gene expression, suggesting modulation of oxidative stress-related protein pathways.

Histopathological examination revealed polynuclear giant cells, sinusoidal dilatation, inflammatory infiltration, fibrosis, cellular degeneration, and focal hepatic necrosis following GLY exposure. QE supplementation mitigated these structural alterations and restored both non-enzymatic antioxidants (GSH, non-protein thiols) and antioxidant enzyme activities (SOD, CAT, GPx) [35].

In addition to its hepatoprotective effects, QE has also been shown to attenuate GLY-induced toxicity in the kidney, suggesting a broader organ-protective role.

Nephroprotective effects of QE (25 or 50 mg/kg/day, via gavage) were observed in a sub-acute male Wistar rat model (25 mg/kg/day of GLY via gavage). After 21 days of GLY exposure, the researchers observed impaired renal function (elevated creatinine, urea, and uric acid), increased kidney weight, and elevated kidney injury molecule-1 (KIM-1). These changes were accompanied by increased oxidative and nitrosative stress, including depletion of GSH, reduced activities of antioxidant enzymes (CAT, SOD, GPx, and GR), increased levels of MDA and nitric oxide, and upregulation of nitric oxide synthase 2 (NOS2) expression.

Glyphosate exposure suppressed antioxidant defense signaling, as shown by reduced Nrf2 protein levels and downregulation of nuclear factor, erythroid 2-like 2 (Nfe2l2), heme oxygenase 1, Cat, Sod2, and Gpx-1. Additionally, it activated inflammatory and apoptotic pathways (increased TLR-4, TNF-α, IL-1β, IL-6, Bax, CYTOCHROME C, and CASPASE-3, with reduced Bcl-2). Histopathological examination confirmed tubular degeneration, glomerular atrophy, leukocyte infiltration, and apoptotic changes.

QE pretreatment attenuated these alterations in a dose-dependent manner (25 and 50 mg/kg/day). Higher doses of QE showed greater potency in restoring renal morphology and function, improving redox balance, suppressing inflammatory signaling, and normalizing markers of apoptosis [50].

Overall, quercetin exerted partial hepatoprotective, nephroprotective, anti-inflammatory, and antioxidant effects, attenuating but not fully reversing GLY-induced oxidative and structural liver damage.

### 3.3. Dietary Supplements and Natural Extracts

#### 3.3.1. Black Seed (*Nigella sativa*)

One study evaluated the protective effects of black seed (*Nigella sativa*, BS) against GLY-induced toxicity in common carp (*Cyprinus carpio*). Fish were pre-treated with varying dietary concentrations of BS for 60 days (0.25, 0.5 and 1% of diet), followed by 14 days of GLY exposure (0.122 mg/dL of water).

BS supplementation attenuated oxidative stress, as evidenced by reduced MDA levels and increased SOD and GPx activities. It also enhanced immune parameters, including lysozyme activity and total immunoglobulin levels. Additionally, BS prevented GLY-induced decreases in serum total protein and albumin, stabilized lipid profiles (cholesterol, LDL, HDL), and reduced liver enzyme activities (ALT, AST, ALP) compared to fish exposed to GLY alone.

The highest dietary concentration (1%) demonstrated greater protective efficacy than lower doses. However, BS supplementation did not fully prevent all adverse effects associated with GLY exposure [51].

Overall, *Nigella sativa* exhibited antioxidant, immunomodulatory, and hepatoprotective effects, although protection against GLY toxicity remained partial.

#### 3.3.2. *Ginkgo biloba*

*Ginkgo biloba* (GB) leaf extract demonstrated protective effects against GLY-induced toxicity in Swiss albino mice. Animals received a single intraperitoneal dose of GLY (50 mg/kg BW), while GB extract (50 or 150 mg/kg BW) was administered orally for eight days (five days prior to and three days following GLY exposure) at varying doses. Protective effects were dose-dependent, with higher doses conferring greater benefit.

GB supplementation attenuated GLY-induced liver and kidney injury, as reflected by reduced AST, ALT, creatinine, and blood urea nitrogen levels, together with improved histopathological findings, including decreased degeneration, necrosis, and fibrosis. The highest GB dose partially restored antioxidant balance, increasing GSH and reducing MDA levels in hepatic and renal tissues, although values remained statistically different from controls.

GLY exposure significantly increased micronucleus frequency and chromosomal aberrations in bone marrow cells. GB extract reduced genotoxic markers, including chromatid breaks, acentric fragments, and chromatid gaps, and was associated with an increased mitotic index, suggesting recovery from GLY-induced chromosomal damage [27].

Collectively, GB extract exhibited dose-dependent antioxidant, organ-protective, and genoprotective effects, although complete normalization was not achieved.

#### 3.3.3. Hawthorn-Leaf Flavonoid

Hawthorn-leaf flavonoid (HF) was evaluated by Dai et al. [52] as a protective agent against GLY-induced toxicity in broiler chickens. Animals were exposed to 25 mg/kg GLY (drunk water), with a subset receiving co-treatment with HF (60 mg/kg in feed) for 29 days.

HF supplementation restored growth performance and feed intake to near-control levels and improved lipid parameters, including triglycerides (TG), total cholesterol (CHOL), and non-esterified fatty acids (NEFA). GLY exposure induced morphological alterations in the ileum, such as mucosal bleeding and reduced microvilli density, which were attenuated by HF. Antioxidant enzyme activities (CAT, SOD, GSH-Px) improved with co-treatment, although MDA levels remained unchanged.

HF also modulated gut microbiota composition, increasing bacterial richness and promoting beneficial genera, including *Lactobacillus*, *Streptococcus*, and *Candidatus Arthromitus*, while limiting the overgrowth of harmful bacteria. However, microbial composition was not fully restored to control profiles.

Metabolomic analysis showed that HF reduced the number of upregulated metabolites and enriched metabolic pathways associated with lipid metabolism (ether lipid metabolism, biosynthesis of unsaturated fatty acids), taurine and hypotaurine metabolism, and neuroactive ligand–receptor interaction. HF decreased toxic bile acids and increased metabolites linked to lipid homeostasis and intestinal health. Additionally, it restored expression of lipid transport proteins (fatty acid-binding protein 2, microsomal triglyceride transfer protein, apolipoprotein A1, apolipoprotein AIV) and attenuated inflammatory signaling (TNF-α, IL-8, interleukin 4 induced 1, NF- κB subunit 1, NF- κB subunit 2, NF-KB inhibitor epsilon). GLY-induced alterations in antioxidant genes (SOD1, SOD3, GPx1) and the tight junction protein Claudin-3 were also reversed, suggesting preservation of gut barrier integrity [52].

HF exerted multi-level protective effects, improving growth performance, antioxidant status, gut microbiota composition, metabolic pathways, and intestinal barrier integrity, although full normalization was not achieved.

#### 3.3.4. Licorice Extract

Licorice (*Glycyrrhiza glabra*, LI) root is recognized for its therapeutic and nutritional applications, which contain several bioactive compounds, including glycyrrhizin, glabridin, liquiritin, glycyrrhizic acid, and glycyrrhetinic acid. Elkattan et al. [53] evaluated the protective effects of LI extract (100, 200, 300 mg/mL, via gavage) against GBH-induced toxicity (Roundup 48%; 24% solution at 1 mL/day, via gavage) in albino male rats under co-administration conditions.

LI supplementation moderated biochemical markers of hepato-renal injury, including ALT, AST, albumin, urea, and creatinine, with the highest dose (300 mg/mL DW) demonstrating the most marked effects. High-dose LI significantly reduced creatinine levels during the first week and delayed their elevation during prolonged exposure compared with lower doses. Elevation of alpha-fetoprotein (AFP) was observed only in the GBH and low-dose LI group (100 mg/mL DW), whereas higher LI doses maintained AFP levels closer to control values.

GBHs’ exposure was associated with reduced thyroid hormone levels (free T3, free T4, T3, and T4). LI supplementation did not fully restore hormonal balance; only partial improvement was noted, particularly in free thyroxine concentrations.

Histopathological analysis exposed that higher LI doses attenuated GBH-induced liver abnormalities, including hydropic degeneration, necrosis, sinusoidal dilatation, fatty changes, and hepatocyte destruction. LI also reduced apoptotic (caspase-3) and inflammatory (TNF-α) markers in a dose-dependent manner. Nevertheless, even the highest dose did not completely normalize all parameters [53].

LI extract exhibited dose-dependent hepato-renal and anti-inflammatory protective effects, although endocrine and molecular parameters were only partially restored.

#### 3.3.5. Linum Usitatissimum Oil

Linum usitatissimum oil (LuO), recognized for its antioxidant properties, was investigated as a potential protective agent against GBH-induced toxicity. The study focused on oxidative stress and hepatic and renal tissue damage in rats exposed to GLY.

Co-supplementation with LuO (0.5 g/kg BW/day, oral way) significantly attenuated GLY-induced (Roundup^®^ TURBO, 269.9 mg/kg BW/day, through drinking water) oxidative stress, reducing MDA, protein carbonyls, and advanced oxidation protein products (AOPP) in liver and kidney tissues. Antioxidant enzyme activities, including SOD, CAT, and glutathione-S-transferase (GST), were also improved.

LuO supplementation mitigated biochemical markers of hepato-renal injury (AST, ALT, ALP, urea, and creatinine) and preserved histological architecture. Although GLY exposure led to reductions in body and organ weights, LuO partially counteracted these effects [54].

LuO exerted antioxidant and organ-protective effects, though the mitigation of systemic GLY toxicity was incomplete.

#### 3.3.6. *Moringa oleifera*

*Moringa oleifera* (MO) leaf extract was evaluated for its protective effects against GLY-induced oxidative stress, inflammation, apoptosis, and organ dysfunction in male Nile tilapia (*Oreochromis niloticus*). Fish received 8 weeks of dietary pre-treatment with MO (200 mg/kg feed) alone or in combination with phytase (0.2 g/kg), followed by a 3-day exposure to Roundup^®^ (30 mg/L of drinking water).

Combined supplementation with MO and phytase significantly improved growth performance parameters, including body weight, weight gain, feed conversion ratio, and specific growth rate, whereas MO alone was associated with reduced growth performance. Pre-treatment with MO, with or without phytase, attenuated elevations in liver and kidney injury markers (AST, ALT, creatinine, blood urea nitrogen), improved lipid profiles, and increased serum total protein and globulin levels.

GBHs exposure induced marked histopathological damage in the gill, liver, and intestinal tissues, which was alleviated by pre-supplementation. At the molecular level, MO (particularly in combination with phytase) upregulated hepatic growth hormone, insulin-like growth factor-1 (IGF-1), and myogenin expression, as well as intestinal ghrelin along with neuropeptide Y (NPY), while downregulating hepatic IGF-binding protein and myostatin expression. MO alone had limited effects on gene expression, except for modulating intestinal ghrelin and NPY.

Additionally, MO supplementation increased SOD activity and reduced expression of inflammatory and apoptotic markers (COX-2 and caspase-3), supporting its antioxidant and anti-inflammatory properties [55].

MO, particularly in combination with phytase, exerted multi-level protective effects, improving growth performance, organ function, and oxidative–inflammatory balance, although responses differed between supplementation strategies.

#### 3.3.7. Propolis Nanoparticles

Propolis supplementation was investigated as a protective strategy against GBH-induced toxicity in Nile tilapia. Fish were exposed to Roundup 48% (0.6 mg/L of water) and propolis (10 g through feed) or propolis nanoparticles (10 g through feed). Co-administration partially mitigated alterations in hematological and biochemical parameters, including white blood cells (WBC) and red blood cells (RBC) counts, ALT, urea, and creatinine levels, although AST activity remained unaffected.

Both propolis and propolis nanoparticles improved immune-related markers, restoring total protein, albumin, globulin concentrations, lysozyme activity, and total immunoglobulin levels. Similar protective effects were observed in oxidative stress parameters, with modulation of MDA, GSH, and antioxidant defenses, as well as normalization of acetylcholinesterase activity, glucose, and cortisol levels.

Notably, propolis nanoparticles demonstrated greater efficacy than crude propolis across most measured variables, in several cases restoring parameters close to control values [56].

To summarize, nanoparticle-form propolis exerted antioxidant, immunomodulatory, and metabolic-protective effects against GBH-induced toxicity.

#### 3.3.8. Selenium

GLY exposure (2 mg GLY /L of water) in Nile tilapia resulted in significant reductions in body weight, weight gain, growth rate, and feeding efficiency, accompanied by altered hematological and biochemical parameters, elevated liver and kidney injury markers, and increased oxidative stress.

Supplementation with selenium yeast (SY) (0.8 g per Kg of feed) during GLY exposure markedly attenuated these effects. Growth performance improved early in the experiment and remained enhanced throughout the 60-day trial. Moreover, cumulative mortality was lower in the SY-supplemented group (~6.7%) compared with GLY-exposed fish (>10%).

SY improved hematological indices, including RBC count, hemoglobin (Hb), and hematocrit (HCT), as well as serum albumin and globulin concentrations. It also mitigated hepato-renal toxicity and modulated oxidative stress markers (MDA, SOD, GPx), particularly in hepatic tissue [57].

Overall, SY supplementation exerted growth-promoting, antioxidant, and hepato-renal protective effects in GLY-exposed Nile tilapia.

#### 3.3.9. Tannic Acid

Abolarin and Owoyele (2024) reported that GBH exposure (Roundup^®^ TURBO 500 mg/kg BW/day, via gavage) increased sensitivity to mechanical, thermal, and chemical nociceptive stimuli in male Swiss albino mice, as reflected by reduced pain thresholds. GBHs elevated prostaglandin E2 (PGE2) levels, decreased dopamine and noradrenaline concentrations, and increased glutamate levels in the brain.

Tannic acid (TA) was evaluated under both pre-treatment and co-administration paradigms (50 mg/kg BW/day, via gavage). Pre-treatment with TA attenuated hyperalgesia and partially restored neurotransmitter equilibrium, whereas co-administration produced more modest effects, particularly on dopamine and glutamate, with no significant impact on noradrenaline.

TA reduced oxidative stress in the prefrontal cortex, lowering MDA and lipid peroxidation (LPO) levels while restoring antioxidant enzyme activities (CAT, SOD, GPx). GBH exposure upregulated pro-inflammatory mediators (NF-κB, TNF-α, interleukin 1 beta (IL-1β), interleukin 6 (IL-6)) and reduced anti-inflammatory cytokines (IL-10, IL-4, TGF-β); these changes were attenuated by TA pre-treatment.

Histological analysis demonstrated that TA improved the structural integrity of the medial prefrontal cortex, reducing neuronal vacuolation and neurodegenerative alterations induced by GBHs. When compared with ascorbic acid (AA), used as a reference antinociceptive and anti-inflammatory agent, TA exhibited comparable—if not slightly stronger—neuroprotective effects, particularly in the pre-treatment setting [58].

TA demonstrated antioxidant, anti-inflammatory, and antinociceptive effects, with greater efficacy observed in the pre-treatment paradigm.

#### 3.3.10. Zinc

Studies investigating zinc (Zn) supplementation as a protective strategy against GBH-induced toxicity have yielded mixed results.

In an early study (2013), Zn pre-treatment was evaluated in Wistar rats exposed to GBHs. Wistar rats were exposed to Zn 50mg/kg BW and Bushfire^®^ (GBH) at 14.4 and 375 mg/kg BW/day via gavage. Histopathological alterations—including degeneration of gastric mucosa, hepatocytes, pancreatic tissue, neurons, renal tubular necrosis, and splenic cell depletion—were observed primarily at the higher GBH dose. Zn supplementation reduced or prevented several of these changes. However, vacuolization was noted in the brains of rats treated with Zn alone, and mild splenic hemosiderosis was observed in Zn-treated animals irrespective of GBH exposure [59].

In a subsequent study (2014) with the same Zn and GBH exposure, co-supplementation with Zn did not significantly modify most biochemical parameters (AST, ALT, urea, creatinine, total protein, globulin, electrolytes). ALP activity showed inconsistent changes across groups, and albumin levels were paradoxically elevated in some GBH-treated animals. These findings suggested a limited biochemical benefit of Zn in this experimental setting [60].

In a chronic exposure model (36 weeks), long-term GBH (Bushfire^®^; 14.4, 375, and 750 mg/kg BW/day, via gavage) administration increased AST, ALT, ALP, and creatinine levels, indicating hepatic and renal injury. Zn pre-treatment (doses: 50 and 100 mg/kg BW/day, via gavage) attenuated elevations in transaminases and reduced creatinine concentrations, although effects on urea were modest and not statistically significant. Electrolyte levels (Na^+^, Cl^−^, K^+^) remained largely unchanged, with some variation in bicarbonate and calcium levels. Histologically, Zn partially mitigated hepatic degeneration but was also associated with mononuclear cell infiltration. Renal lesions induced by GBHs were not fully prevented [36].

In a subchronic model (16 weeks), researchers used Zn at a 50 mg/kg BW/day concentration and 187.5 or 375 mg/kg BW of GBH (Gobara^®^) via gavage to research their thesis. Zn co-administration reversed GBH-induced disturbances in calcium, vitamin D3, and parathormone (PTH) levels, suggesting modulation of endocrine responses. GBHs caused structural damage in the parathyroid gland, femoral bone, and skeletal muscle, whereas Zn co-treatment prevented these lesions [61].

Zn supplementation demonstrated partial protective effects in certain biochemical and histopathological parameters; however, findings were inconsistent across studies, and some adverse or neutral effects were also reported.

Collectively, dietary supplements and plant-derived extracts demonstrated predominantly antioxidant, anti-inflammatory, and organ-protective effects across experimental models, although the magnitude and consistency of protection varied depending on the compound, dose, and exposure paradigm.

### 3.4. Humic Substances

Humic substances, particularly humic acid (HA), have been investigated for their potential to neutralize GLY toxicity and reduce tissue residues. In chickens, HA supplementation reduced GLY accumulation in tissues [69].

In broiler breeder roosters, GLY exposure (Roundup PRO^®^ 1.25, 2.50 mL/kg of feed ~ 12–31 mg GLY/kg BW/day) significantly impaired sperm count, motility, viability, and semen volume. HA co-administration (HA 30g/kg of feed) reversed these alterations, in some parameters restoring values to or above control levels. After a 4-week recovery period without GLY or HA exposure, sperm quality no longer differed between groups. GLY-induced vacuolation of seminiferous tubules was attenuated by HA, while body weight remained unaffected. Notably, dietary HA reduced GLY levels in feed by 13% compared to control and by 91% relative to GLY-treated feed alone, and fecal GLY residues were undetectable in the HA co-treated group. Plasma testosterone levels were elevated during exposure in both HA and GLY groups, but normalized after the recovery period; androgen receptor expression was restored only in HA-supplemented roosters [62].

In adult zebrafish (Danio rerio), HA was evaluated for its capacity to preserve hepatic proteomic integrity. Zebrafish were kept in water containing Zn 20 at mg/L and GLY at 100 μg/L, AMPA at 100 μg/L, or its mixture (50 + 50 μg/L. GLY and its metabolite AMPA exhibited synergistic effects, disrupting oxidative phosphorylation, apoptosis, necroptosis, DNA repair, immune signaling, and protein translation pathways. HA alleviated proteomic alterations induced by GLY and by the GLY+AMPA mixture; however, it unexpectedly exacerbated changes associated with AMPA alone. Two proteins involved in stress and DNA repair regulation (RuvB-like2 and eukaryotic translation initiation factor 2 subunit 1) were downregulated across treatment groups [63].

HA demonstrated residue-reducing and partially protective effects against GLY-induced reproductive and proteomic alterations, although its interaction with AMPA appeared more complex.

### 3.5. Hormonal Modulators

#### Melatonin

Hormonal Modulators Melatonin (ME), beyond its circadian role, has been investigated as a potential protective agent against GLY and GBH-induced toxicity.

In vitro exposure of mouse oocytes to low concentrations of Roundup ^®^ (In vitro: 0.00001%, 0.00005%, and 0.00025%) disrupted meiotic progression, as evidenced by reduced polar body extrusion, spindle disorganization, chromosome misalignment, and impaired sperm-binding capacity. Co-treatment with ME (1, 10, and 100 µM) restored meiotic competence and fertilization rates while reducing ROS generation and apoptosis markers to near-control levels. In vivo, environmentally relevant GBH exposure (0.0005% Roundup^®^ solution through drinking water) reduced early embryo cleavage rates and induced endocrine disturbances; ME supplementation (0.15 or 1.5 mg/kgBW/day, through drinking water) improved 2-cell embryo formation and normalized G protein-coupled estrogen receptor (GPER)/G protein-coupled receptor 30 expression, while preserving downstream MAPK/ERK1/2 signaling. Comparable protective effects were observed with a GPER antagonist (G15), suggesting receptor-mediated mechanisms [64].

In a renal model, ME attenuated GLY-induced tubular injury, reducing neutrophil gelatinase-associated lipocalin, β2-microglobulin, and albumin levels, and improving histopathological features. ME also modulated endoplasmic reticulum stress pathways (Bip, ATF6, PERK) and suppressed renal pyroptosis, as reflected by reduced NLRP3 activation, caspase-1 cleavage, IL-1β maturation, and gasdermin D expression. In this model, male ICR mice were given Me at 10 mg/kg BW/day via intraperitoneal injection, and Roundup^®^ at 100 mg/kg⋅BW/day via drinking water [34].

In roosters, GLY (GLY 45521 at 200 mg/kg in feed) exposure impaired testosterone synthesis by inducing mitochondrial dysfunction and activating Parkin-dependent mitophagy, accompanied by Leydig cell damage and deterioration of seminiferous tubule structure. Co-administration of ME (10 mg/kg BW/day of Me, via intraperitoneal injection) restored mitochondrial function, inhibited excessive mitophagy, improved sperm parameters, and reduced oxidative stress in testicular tissue [65].

Collectively, ME demonstrated multi-organ protective effects, including antioxidant, anti-apoptotic, anti-pyroptotic, and mitochondria-stabilizing, with evidence supporting receptor-mediated and intracellular signaling mechanisms.

### 3.6. Other Natural Compounds

#### 3.6.1. Trehalose

Trehalose (Tre), a naturally occurring disaccharide with antioxidant and anti-inflammatory properties, was evaluated as a protective agent in roosters chronically exposed to 200 mg/kg of GLY in feed (120 days). Tre co-administration (5 g/kg in feed) significantly reduced GLY levels in serum and liver tissue, suggesting a potential role in enhancing GLY clearance or limiting tissue accumulation.

GLY exposure induced liver injury, characterized by histological disorganization (cellular swelling, cytoplasmic vacuolation), elevated transaminases (AST, ALT), ALP, γ-GT, and dyslipidemia (altered TG, TC, LDL-C, HDL-C levels). Tre supplementation mitigated these changes, improving hepatic architecture and partially normalizing biochemical parameters. OS markers were also modulated: GLY increased MDA and reduced total antioxidant capacity (T-AOC), SOD, CAT, and GSH-Px, whereas Tre restored antioxidant defenses toward control values. Mechanistically, Tre activated the Nrf2 pathway, increasing Nrf2, HO-1, and NAD(P)H quinone dehydrogenase 1 (NQO1) expression, reducing Keap1 levels, and suppressing NLRP3 inflammasome activation (caspase-1 and IL-1β) [66].

In a separate study focusing on male reproductive toxicity at the same exposure concentration, Tre reduced GLY accumulation in serum and testicular tissue, improved testis size, and mitigated structural alterations in seminiferous tubules. GLY-induced increases in ROS and MDA were attenuated, with concomitant restoration of antioxidant enzymes. Consistent with hepatic findings, Tre activated Nrf2 signaling and restored HO-1 and NQO1 expression in testicular tissue [67].

Tre demonstrated antioxidant and anti-inflammatory effects across hepatic and reproductive models, largely mediated through activation of the Nrf2 pathway and suppression of inflammasome signaling.

#### 3.6.2. *Xylopia aethiopica*

*Xylopia aethiopica* (XYPAE), a plant extract with reported antioxidant and anti-inflammatory properties, was evaluated in Wistar rats exposed to GBHs. Rats were treated with XYPAE 126.49 mg/kg BW/day, and Roundup ^®^ 150 mg/kg BW/day, orally. GBH exposure induced oxidative stress, neuroinflammation, and structural brain alterations. Co-supplementation with XYPAE improved antioxidant status (SOD, CAT, GSH, GSH-Px) and modulated brain function markers, including acetylcholinesterase and butyrylcholinesterase activities.

Pro-inflammatory mediators elevated by GBHs (TNF-α, *C*-reactive protein, IL-6) were reduced following XYPAE treatment. Histopathological analysis demonstrated attenuation of GBH-induced cortical, cerebellar, and hippocampal damage, including reduced neuronal degeneration, preservation of cellular architecture, and decreased vacuolation.

When compared with vitamin C, XYPAE showed broadly similar neuroprotective effects; however, vitamin C exerted stronger downregulation of apoptotic and inflammatory markers such as caspase-3, p53, and COX-2 [68].

In summary, XYPAE demonstrated antioxidant and anti-inflammatory neuroprotective effects, although its efficacy appeared slightly lower than that of vitamin C in modulating apoptosis-related pathways.

## 4. Discussion

Significant concerns have been raised regarding the potential toxicity of GLY and GBHs in both humans and animals. Numerous studies have linked exposure to these herbicides with oxidative stress (OS), inflammation, organ toxicity, endocrine disruption, metabolic imbalance, and genotoxicity [70]. The present narrative review addressed the lack of a consolidated synthesis of experimental mitigation strategies for GLY-related toxicity. Experimental evidence suggests that a broad range of compounds may attenuate GLY- and GBH-induced adverse effects [71,72]. Despite heterogeneity across models and study designs, most reports concentrate on shared biological mechanisms, primarily involving modulation of oxidative stress, inflammatory signaling, and cellular stress response pathways [73].

The available evidence is also complicated by substantial heterogeneity across experimental models. The reviewed studies differ considerably in the species used, exposure conditions, doses, routes of administration, and exposure duration. In addition, some studies investigated pure glyphosate, whereas others used commercial glyphosate-based herbicide formulations. High variability makes direct comparisons between studies difficult, limiting the strength of the resulting conclusions.

Restoration of redox homeostasis, reflected by normalization of antioxidant enzyme activity and attenuation of lipid and DNA oxidative damage, was shown to be the most prominent protective mechanism. These antioxidative effects were observed across multiple classes of compounds, including vitamins, classical antioxidants, dietary supplements, and selected natural products [27,32,33,35,38,39,42,43,44,47,48,49,51,54,55,56,57,58,64,66,67,68]. However, those results were formed from in vitro or in vivo animal studies. They may not fully capture the reflection of human physiology. While oxidative stress appears to represent a common pathway of GLY and GBHs toxicity, its role in humans requires cautious interpretation.

Importantly, not all interventions demonstrated consistent protective efficacy, and in several models, the observed benefits were limited to selected biomarkers without full functional restoration.

Inflammatory signaling constitutes a second major mechanistic axis. Several micronutrients and natural compounds suppressed GLY and GBH-induced activation of NF-κB and NLRP3 inflammasome pathways, with reductions in pro-inflammatory cytokines such as TNF-α, IL-1β, and IL-6 [38,52,53,55,58,66,67,68]. Nonetheless, some studies reported limited or inconsistent modulation of inflammatory mediators, suggesting variability related to experimental conditions, compound selection, dosing, or timing of intervention.

Emerging evidence also implicates mitochondrial dysfunction and endoplasmic reticulum stress in GLY/GBH toxicity. Selected compounds demonstrated protective effects through stabilization of mitochondrial integrity, inhibition of Parkin-dependent mitophagy, and modulation of GPER–MAPK/ERK1/2 signaling pathways [34,64,65]. These findings support the hypothesis that mitochondrial and stress response networks may represent critical vulnerability nodes in glyphosate toxicity and potential targets for intervention.

The available evidence indicates that mitigation of GLY/GBH toxicity is unlikely to depend on modulation of a single pathway, but rather on coordinated regulation of oxidative, inflammatory, and cellular stress response networks. The potential synergistic or antagonistic interactions between protective compounds remain largely untested.

A recurring pattern across compound classes was convergence on redox-sensitive transcriptional regulation, particularly involving Nrf2-mediated antioxidant responses and NF-κB-dependent inflammatory cascades. Mitochondrial integrity may be a key vulnerability point in the toxicity of GLY and GBHs and a suitable target for mitigation techniques.

### 4.1. Translational Application

Given several methodological limitations, the translational application of these data should be handled with caution. All available evidence derives from animal models or in vitro systems, which do not completely capture the complexity of human physiology or real-world exposure scenarios. Many study designs employed exposure conditions, dosages, and durations that exceed environmentally or dietarily relevant human exposure levels, thereby restricting clinical relevance. The discrepancy between experimental exposure paradigms and real-life chronic low-dose human exposure remains an essential gap in current mitigation research [31].

Interpretation of the reported protective effects should also consider whether the underlying GLY/GBH exposure approximates human-relevant levels. In the current European Union assessment, the acceptable daily intake (ADI) for glyphosate is 0.5 mg/kg body weight/day, and the acute reference dose (ARfD) is 1.5 mg/kg body weight [31]. Findings obtained under very high-dose conditions should be interpreted primarily as mechanistic evidence rather than as direct approximations of typical human exposure scenarios. Additional caution is warranted for studies using non-dietary or otherwise non-physiological routes of administration, as such designs may exaggerate both toxic and protective effects, thereby reducing external validity [74].

An additional source of heterogeneity derives from the use of pure GLY versus commercial GBHs. Although GLY is the active ingredient in GBHs, formulations differ substantially in toxicological profiles, with adjuvants considerably increasing its toxicity [75]. Findings obtained under pure GLY exposure should therefore not be directly extrapolated to GBH formulations [12]. Variability in formulation types and inconsistent reporting of composition additionally hinder cross-study comparability. A recent meta-analytical assessment of animal studies highlighted the need to distinguish between pure GLY and GBHs, including dose, concentration, and the presence of adjuvants in commercial formulations, which are important sources of variability and may affect the interpretation of toxicity outcomes [76].

Future research would benefit from standardized experimental protocols, including clear reporting of formulation type, dose equivalence, and exposure length. Chronic low-level exposure models that better reflect typical human exposure patterns may improve translational reliability when assessing potential mitigation strategies.

### 4.2. Safety Considerations and Dose Relevance

Several reviewed compounds in this review are commonly available as dietary supplements, and some also have established clinical use. However, their protective effects cannot be assumed in the context of GLY/GBH toxicity mitigation. For example, NAC is an approved antidote for acetaminophen overdose and is also used in respiratory medicine in high doses, whereas prolonged-release ME is used clinically for insomnia at a fixed dose of 2 mg once daily in older adults [77,78,79]. In experimental studies, the administered amount of a protective compound is not always directly equivalent to a defined human therapeutic dose, particularly when, in some studies, intake depends on consumption of feed or drinking water [34,62,64]. This distinction is important because both NAC and melatonin may be associated with adverse effects or clinically relevant interactions, as with other dietary supplements [77,80]. Although early experimental findings suggest that melatonin may attenuate GLY/GBH-related injury, it should be interpreted carefully, given its role as an endogenous hormone with systemic endocrine and chronobiotic functions [81]. This stresses the importance of adopting a holistic perspective.

Importantly, long-term safety, off-target effects, and drug–supplement interactions were rarely evaluated in the reviewed studies, further limiting conclusions regarding real-life applicability.

### 4.3. Risk of Bias and Limitations of Primary Evidence

The present findings are subject to numerous probable biases in the primary literature. The experimental models used across studies are highly heterogeneous, encompassing different species, exposure paradigms, dosages, formulations, and outcome measures. This limits formal comparability and precludes quantitative synthesis. Potential publication bias favoring positive mitigation findings cannot be excluded.

Further mechanistic research is appropriate to better delineate shared and compound-specific pathways of GLY and GBH toxicity and to identify biologically plausible targets for mitigation.

From a regulatory perspective, recent European Union (EU) evaluations distinguish hazard classification from risk assessment. In 2022, the European Chemicals Agency (ECHA) concluded that the available evidence did not support classification of glyphosate as carcinogenic, mutagenic, or reprotoxic (CMR), while continuing classification for serious eye damage and aquatic toxicity [82]. In 2023, the European Food Safety Authority (EFSA) peer review did not identify critical areas of concern for glyphosate use under current regulatory conditions, although uncertainties and data gaps were acknowledged, notably regarding formulation-specific effects (GBHs versus pure GLY) [31].

Importantly, adjuvants present in commercial GBH formulations may substantially increase overall toxicity compared with pure glyphosate [70]. Conclusions derived from studies using GBHs should not be generalized to pure GLY. The inconsistent use of pure GLY versus various commercial formulations adds further complexity to cross-study comparison and translational interpretation. Additionally, a considerable proportion of studies use high doses or non-dietary routes of administration that differ from typical human exposure patterns, thereby limiting external validity.

## 5. Conclusions

Available experimental evidence indicates that diverse classes of compounds may attenuate GLY-related toxicity through antioxidant, anti-inflammatory, and mitochondrial-stabilizing mechanisms, illustrating shared biological pathways as potential targets for intervention.

Current evidence indicates that OS and interconnected inflammatory–mitochondrial signaling represent central convergence points of GLY and GBH toxicity and may constitute primary targets for future mitigation research.

Nevertheless, substantial methodological heterogeneity, formulation variability, and limited human relevance of many exposure models constrain direct translation of these data into clinical or public health practice. Although the mechanistic plausibility of mitigation strategies is supported by preclinical data, their efficacy and safety in humans remain uncertain.

Future research should prioritize standardizing experimental protocols, conducting formulation-specific analyses, and developing chronic low-dose exposure models. Robust conclusions regarding protective strategies against glyphosate exposure will require harmonized and translationally relevant study designs.

## Figures and Tables

**Table 1:** Summary of Protective Effects of Various Substances Against Glyphosate-Induced Toxicity.

Ref.	First Author (Year)	Type of Study	Animal/Organism	Protective Substance(s) (Doses)	GLY/GBH (Dose)	Length of Exposure (GLY & Protective)	Timing of Protective Administration	Mechanism/Key Biomarkers Studied	Effect on Glyphosate Toxicity
[38]	Ngatuni et al. (2022)	In vivo	Swiss albino mice	VitB12 (10 mg/kg BW/day administered orally)	GBH (375 mg/kg BW/day administered orally)	56 days total; some mice received vitB12 for 7 days before continuing with vitB12+GLY for remainder time	Co-administration or pre-treatment and co-administration	Hematological parameters (RBCs, WBCs, platelets), GSH, TNF-α, IL-10, bilirubin, ALT/AST, organ histopathology	Stabilized blood cells, improved oxidative status, lowered inflammation, protected liver function against GLY
[39]	Gehin et al. (2005)	In vitro	*Human keratinocytes* cells	VitC or/and VitE (100 and 200 µM)	Roundup^®^ 3plus or GLY (10, 12.5, 15, 17.5, 20, 22, and 25 mM)	24–42 h cell culture	Pre-treatment and co-incubation	ROS generation, cell viability, optimization of antioxidant ratio	VitC/E reversed oxidative damage & cell death from Roundup/GLY
[32]	Gehin et al. (2006)	In vitro	*Human keratinocytes* cells	VitC or VitE (100 µM)	Roundup^®^ 3plus or GLY (10, 12.5, 15, 17.5, 20, 22, and 25 mM)	24–48 h in cell culture	Co-incubation	Lipid peroxidation, SOD, CAT, GPx, GSH/GSSG, cell viability	VitC/E restored antioxidant status and reduced cytotoxicity
[40]	Bhardwaj et al. (2019)	In vitro	Caprine granulosa cells	VitC or VitE (50 and 100 µM)	Roundup^®^ (0.1, 2.0, and 4.0 mg/mL)	24, 48, and 72 h in cell culture	Co-incubation	Caspase-3, ROS, cell viability, apoptotic morphology	VitC/E reduced apoptosis & oxidative damage in granulosa cells
[41]	Bhardwaj et al. (2022)	In vitro	Caprine granulosa cells	VitC and VitE (50 and 100 µM)	Roundup^®^ (0.1, 2.0, and 4.0 mg/mL)	24, 48, and 72 h in cell culture	Co-incubation	DNA damage (comet assay), apoptotic markers (caspase, TUNEL)	VitC/E decreased genotoxic & apoptotic damage from GLY
[42]	Fréville et al. (2024)	In vitro	Chicken granulosa cells	VitE (1 mg/L)	Gallup Super 360 (0.036, 0.36, 3.6 and 36 mg GLY eq/L)	48 h in cell culture	Co-incubation	Cell viability, proliferation, oxidative stress (ROS, SOD, CAT, MDA), and steroidogenesis (progesterone secretion).	VitE restored progesterone secretion, reduced intracellular ROS, increased antioxidant enzyme activities, and decreased MDA.
[43]	Mehr et al. (2024) Nie ma daty	In vivo	Female BALB/c mice	Vitamin E (100 mg/kg BW/day, via intraperitoneal injection)	Glyphosate (250 or 500 mg/kg BW/day via gastric gavage)	7 days	Co-administration (Vit E given 2 h after GLY)	MDA, CAT, GSH, estrogen, progesterone, gene expression (Bax, Bcl-2, caspase-3, -9, StAR, 3β-HSD)	VitE reduced oxidative stress, normalized hormone levels, and reversed apoptosis and steroidogenic gene expression changes
[44]	Güngör et al. (2024)	In vivo	Male Wistar Albino rats	Hesperidin (Vitamin P, 100 mg/kg BW/day, via gastric gavage)	Knockdown 48 SL (787.85 mg/kg BW/day, via gastric gavage)	56 days	Co-administration	Sperm motility, DNA damage (COMET), MDA, GSH, TAS, TOS, Histopathology	Hesperidin improved sperm quality, reduced oxidative stress, DNA damage, and testicular histopathology alterations
[33]	Türkmen et al. (2025)	In vivo	Wistar Rats	Chlorogenic Acid (12.5, 25, 50 mg/kg BW/day, via gavage)	Knock-out^®^ (800 mg/kg BW/day, via gavage)	49 days	Pre-treatment (1 h before GBH)	MDA, GSH, SOD, CAT, 8-OHdG, AST, ALT, ALP, Histopathology	CGA dose-dependently reversed oxidative stress, DNA damage, and tissue histopathology
[45]	Mutluay et al. (2025)	In vivo	Adult male ICR mice	Coenzyme Q10 (200 mg/kg BW/day, via gavage)	Roundup^®^ (500 mg/kg BW/day, via gavage)	35 days	Co-administration	Testicular histology, sperm quality and mitochondrial ROS, hormones (testosterone, LH), oxidative stress (MDA, TOS, OSI, GSH, TAS), Nrf2/Keap1/HO-1, TLR4/NF-κB, Bax/Bcl-2	CoQ10 improved sperm and testicular outcomes, restored redox balance, normalized signaling pathways, reduced apoptosis, and improved hormonal profile
[46]	Dogan et al. (2025)	In vivo	Adult male rats	Eugenol (50 or 100 mg/kg, via gavage)	Roundup^®^ (150 mg/kg, via gavage)	7 days	Co-administration	Histopathology; oxidative stress (MDA, GSH, SOD, CAT, GPx); PI3K/AKT signaling; ER stress-, RAGE-, and NLRP3-related gene expression; Western blot for Bax, Bcl-2, caspase-3, Beclin-1, NF-κB, TNF-a, Keap1, Nrf2	EUG (especially 100 mg/kg) reduced oxidative/ER stress, inflammation, and apoptosis, and improved testicular histology via PI3K/AKT pathway modulation
[47]	Turkmen et al. (2019)	In vivo	Wistar albino rats	*N*-acetylcysteine (160 mg/kg BW/day, via gastric gavage)	Knockdown 48SL (GLY ~ 375 mg/kg BW/day, via gastric gavage—1/10 LD50)	8 weeks	Co-administration	Blood/tissue GSH, MDA, SOD, CAT, histopathology (liver, kidney, heart, brain)	NAC restored antioxidant levels & protected multiple organs from GBH-induced lesions
[48]	Hashim et al. (2021)	In vivo	Adult male albino rats	*N*-acetylcysteine (160 mg/kg BW/day, via gavage)	Roundup^®^ (0.8503 mL/kg = GLY ~ 375 mg/kg BW/day, via gavage)	6 weeks	Co-administration	Serum ALT/AST, MDA, TAC, Nrf2 expression, histopathology, PCNA & caspase-3 immunostaining	NAC partially reversed oxidative stress & liver injury from GLY; improved histology & lowered apoptotic markers
[49]	Bhardwaj et al. (2022)	In vitro	Caprine testicular germ cells	*N*-acetylcysteine (100 µM)	Roundup^®^ (10, 12.5, 15, 17.5, 20, 22, and 25 mM)	24, 48, and 72 h	Co-incubation	Lipid peroxidation, SOD, CAT, GST, apoptotic changes	NAC rescued testicular cells, decreased oxidative stress & apoptosis
[35]	Soudani et al. (2019)	In vivo	Adult male Wistar rats	Quercetin (20 mg/kg BW/day, via gavage)	GLY (50 mg/kg BW, intraperitoneal injection every 2 days)	15 days	Co-administration	MDA, H_2_O_2_, SOD, CAT, GPx, advanced oxidation proteins, metallothionein genes, histopathology	Quercetin restored antioxidant balance, reduced liver damage, and normalized metallothionein gene expression
[50]	Albrakati (2025)	In vivo	Male Wistar rats	Quercetin (25 or 50 mg/kg/day, via gavage)	Glyphosate (25 mg/kg/day, via gavage)	21 days	Co-administration	Renal function and KIM-1; oxidative stress (MDA, NO, GSH); antioxidant enzymes and genes (CAT, SOD, GPx/GR; Cat, Sod2, GPx-1); Nrf2/Nfe2l2/Hmox-1; inflammatory markers (TNF-a, IL-1b, IL-6, TLR4, NOS2); apoptosis (Bax, cytochrome c, caspase-3, Bcl-2); histology	Quercetin dose-dependently attenuated nephrotoxicity, restoring antioxidant defenses, suppressing inflammation and apoptosis, and improving renal histopathology (higher dose more effective)
[51]	Yousefi et al. (2021)	In vivo	Common carp (Cyprinus carpio)	Black seed (Nigella sativa) (0.25, 0.5 and 1% of diet)	GLY (0.122 mg/dL of water)	60 days black-seed feeding + 14 days GLY exposure	Pre-treatment	Immune depression (lysozyme, Ig), oxidative stress (MDA, SOD, GPx), metabolic function, stress hormones	Prevented immune/metabolic dysregulation, lowered oxidative stress, improved fish health under GLY
[27]	Çavuşoğlu et al. (2011)	In vivo	Swiss albino mice	Ginkgo biloba L. leaf extract (50 or 150 mg/kg BW, orally)	Roundup Ultra-Max (50 mg/kg BW single dose by intraperitoneal injection)	8 days	Pre-treatment, co-administration, and post-treatment	AST, ALT, BUN, creatinine, MDA, GSH; micronucleus frequency, chromosomal aberrations, histopathology (liver/kidney)	Reduced genotoxicity, oxidative injury, and organ damage from GLY
[52]	Dai et al. (2024)	In vivo	Male Arbor Acres broiler chicks	Hawthorn-leaf flavonoid (60 mg/kg, through feed)	GLY (25 mg/kg, through drinking water)	29 days	Co-administration	Microbiota (16S), antioxidant enzymes, lipid profile, inflammation	HF restored microbiota balance, improved gut barrier, and reduced inflammation
[53]	Elkattan et al. (2024)	In vivo	Adult male albino rats	Licorice extract (100, 200, 300 mg/mL, via gavage)	Roundup 48% (24% solution at 1 mL/day, via gavage)	3 weeks	Co-administration	ALT, AST, albumin, creatinine, AFP, thyroid hormones, TNF-α, caspase-3, histopathology	Higher licorice dose strongly ameliorated hepatic/renal damage & inflammation from GLY
[54]	Djaber et al. (2020)	In vivo	Wistar rats	Linum usitatissimum oil (0.5 g/kg BW/day, oral way)	Roundup^®^ TURBO (269.9 mg/kg BW/day, through drinking water)	30 days	Co-administration	Serum AST, ALT, ALP, creatinine, MDA, PCO, SOD, CAT, GST, histopathology	LuO alleviated hepatic/renal toxicity from Roundup, reduced oxidative injury, and improved organ morphology
[55]	Elahwl et al. (2025)	In vivo	Nile Tilapia	Moringa oleifera leaf extract (200 mg/kg) ± phytase enzyme (0.2 g/kg)	Roundup^®^ (30 mg/L of water).	8 weeks	Pre-treatment	Growth markers, antioxidant capacity, apoptosis, and inflammation	MOLE improved growth, antioxidant status, reduced apoptosis and inflammation
[56]	Abdelmagid et al. (2022)	In vivo	Nile tilapia	Propolis (10 g through feed) or propolis nanoparticles (10 g through feed)	Roundup 48% (0.6 mg/L of water)	2 and 4 weeks	Co-administration	Immune parameters (lysozyme, immunoglobulins), RBC/WBC, MDA, GSH, stress biomarkers	Propolis nanoparticles improved fish immunity, lowered oxidative stress, and prevented organ impairment from GLY
[57]	Hassan et al. (2022)	In vivo	Nile tilapia	Selenium yeast (0.8 g per Kg of feed)	GLY (2 mg/L of water)	60 days	Co-administration	Growth, RBC/WBC, hepatic/renal function, MDA, SOD, GPx, mortality	Se-yeast improved growth, lowered oxidative stress & organ injury from GLY ± malathion
[58]	Abolarin and Owoyele (2024)	In vivo	Male Swiss albino mice	Tannic acid (50 mg/kg BW/day, via gavage)	Roundup^®^ TURBO (500 mg/kg BW/day, via gavage)	48 days (with behavioral pain tests around days 46–48)	Pre-treatment (7days) or co-administration	Inflammatory mediators (PGE2, TNF-α, NF-κB), oxidative stress (MDA, SOD, GPx), pain behavior, and neural histology	TA reduced inflammation, oxidative damage, and neurodegeneration from GBH
[59]	Tizhe et al. (2013)	In vivo	Wistar rats	Zinc (50 mg/kg BW, via gavage)	Bushfire^®^ (14.4 and 375 mg/kg BW/day, via gavage)	8 weeks	Pre-treatment	Histopathology: stomach, liver, kidney, brain, pancreas, spleen	Zn alleviated but did not fully prevent organ degeneration from high-dose GLY
[60]	Tizhe et al. (2014)	In vivo	Wistar rats	Zinc (50 mg/kg BW/day, via gavage)	Bushfire^®^ (14.4 and 375 mg/kg BW/day, via gavage)	8 weeks	Co-administration	Serum ALT, AST, ALP, albumin, creatinine, electrolytes	Zn partially preserved normal hepatic/renal function, but protection varied by GLY dose
[36]	Tizhe et al. (2020)	In vivo	Wistar rats	Zinc (50 and 100 mg/kg BW/day, via gavage)	Bushfire^®^ (14.4, 375, and 750 mg/kg BW/day, via gavage)	36 weeks	Co-administration	Serum AST, ALT, ALP, creatinine, Ca^2+^, HCO_3_^−^, histopathology of liver/kidney	Zn decreased serum enzyme elevations & partially reduced organ damage, but did not fully prevent chronic toxicity
[61]	Tizhe et al. (2024)	In vivo	Wistar Rats	Zinc chloride (50 mg/kg BW/day, via gavage)	Gobara^®^ (187.5 and 375 mg/kg BW, via gavage)	16 weeks	Co-administration (Zn was given 1 h before GBH every day)	Calcium, Vitamin D, Parathormone, Histology (bone, muscle, parathyroid)	Zn prevented hypocalcemia, hypovitaminosis D, and tissue damage
[62]	Jarrell et al. (2021)	In vivo	Broiler breeder roosters	Humic acids (30g/kg of feed po.)	Roundup PRO^®^ (1.25, 2.50 mL/kg of feed po. ~ 12–31 mg GLY/kg BW/day)	18 weeks	Co-administration	Sperm quality (count, motility, viability), testis morphology, testosterone, feed/fecal GLY residue	HA adsorbed glyphosate, improved fertility markers, lowered testicular damage & GLY residues in feed/feces
[63]	Morozov and Yurchenko (2024)	In vivo	Zebrafish (*Danio rerio*)	Humic Acid (20 mg/L in water)	Glyphosate (100 μg/L), AMPA (100 μg/L), or mixture (50 + 50 μg/L in water)	28 days (14 days HA + 14 days GLY/AMPA)	Pre-treatment and co-administration	Liver proteomics, stress proteins (e.g., EIF2S1, RuvB-like 2), necroptosis/apoptosis pathways, oxidative phosphorylation, steroid metabolism	HA modulated the effects of GLY/AMPA: mitigated glyphosate-induced changes, enhanced AMPA toxicity alone, but reduced toxicity when co-exposed
[64]	Cao et al. (2021)	In vivo & in vitro	Female ICR mice (oocytes)	Melatonin (in vivo: 0.15, and 1.5 mg/kg BW/day, through drinking water; in vitro: 1, 10, and 100 µM)	Roundup ^®^ (in vivo: 0.0005%, through drinking water; In vitro: 0.00001%, 0.00005%, and 0.00025%)	In vivo: 21 days In vitro: 15hours	Co-administration and co-incubation	Oocyte meiotic progression, ROS, apoptosis (TUNEL), GPER/ERK signaling, fertilization	ME rescued oocyte from GLY-induced oxidative stress & apoptosis, improved fertilization
[34]	Ding et al. (2022)	In vivo	Male ICR mice	Melatonin (10 mg/kg BW/day, via intraperitoneal injection)	Roundup ^®^ (100 mg/kg⋅BW/day, through drinking water)	12 weeks	Co-administration	Kidney function (urea, creatinine), ER stress (BiP, PERK, ATF6), pyroptosis (NLRP3, c-caspase-1), histopathology	ME improved renal function, reduced ER stress & pyroptosis under GLY+hard water
[65]	Ren et al. (2024)	In vivo	Hy-line brown roosters	Melatonin (10 mg/kg BW/day, via intraperitoneal injection)	GLY 45521 (200 mg/kg in feed)	24 weeks	Co-administration, ME for 4 weeks prior to the end of the experiment	Mitochondrial function (Parkin-dependent mitophagy), testosterone, testis histopathology	ME prevented abnormal mitophagy & restored testosterone production in GLY-exposed roosters
[66]	Lian et al. (2023)	In vivo	Hy-line brown roosters	Trehalose (5 g/kg in feed)	GLY 45521 (200 mg/kg in feed)	120 days	Co-administration	Assessed serum hepatic injury markers (AST, ALT, γ-GT), lipid profiles (TC, TG, LDL-C, HDL-C), hepatic MDA, antioxidant enzyme activities, Nrf2 signaling, and NLRP3 inflammasome activation.	Trehalose boosted hepatic antioxidant capacity, suppressed inflammasome activation, and prevented steatosis
[67]	Chen et al. (2022)	In vivo	Hy-line brown breeder roosters	Trehalose (5 g/kg in feed)	GLY 90479 (200 mg/kg in feed)	6 month	Co-administration	Testicular oxidative stress (MDA, SOD, CAT, GSH-Px), Nrf2/HO-1, histopathology	Trehalose alleviated testicular oxidative damage & apoptosis from GLY
[68]	Adewale et al. (2023)	In vivo	Male Wistar rats	*Xylopia aethiopica* extract (126.49 mg/kg BW/day, orally)	Roundup ^®^ (150 mg/kg BW/day, orally)	1 week	Co-administration	Brain oxidative stress (SOD, CAT, GPx), inflammation (TNF-α, IL-6, CRP), immunohistochemistry (caspase-3, COX-2, p53), histology of cortex/cerebellum/hippocampus	XYPAE decreased neuroinflammation, reduced oxidative stress & neuronal damage from GLY

Abbreviations: Ref.—reference number; GLY—glyphosate; GBH—glyphosate-based herbicide; AMPA—aminomethylphosphonic acid; BW—body weight; i.p.—intraperitoneal injection; p.o.—per os/oral administration; LD50—median lethal dose; VitB12—vitamin B12; VitC—vitamin C; VitE—vitamin E; CGA—chlorogenic acid; CoQ10—coenzyme Q10; EUG—eugenol; NAC—*N*-acetylcysteine; LuO—Linum usitatissimum oil; TA—tannic acid; ME—melatonin; HA—humic acid; XYPAE—*Xylopia aethiopica* extract; Zn—zinc; RBC—red blood cells; WBC—white blood cells; BUN—blood urea nitrogen; ALT—alanine aminotransferase; AST—aspartate aminotransferase; ALP—alkaline phosphatase; γ-GT—gamma-glutamyl transferase; CK-MB—creatine kinase-MB; cTn-I—cardiac troponin I; KIM-1—kidney injury molecule-1; MDA—malondialdehyde; LPO—lipid peroxidation; OS—oxidative stress; ROS—reactive oxygen species; SOD—superoxide dismutase; CAT—catalase; GPx—glutathione peroxidase; GSH—glutathione; GSSG—oxidized glutathione; GSSG-Red—glutathione reductase; GST—glutathione S-transferase; TAC—total antioxidant capacity; TAS—total antioxidant status; TOS—total oxidant status; OSI—oxidative stress index; 8-OHdG—8-hydroxy-2′-deoxyguanosine; NO—nitric oxide; NOS2—nitric oxide synthase 2; TNF-α—tumor necrosis factor-alpha; NF-κB—nuclear factor kappa B; IL-10—interleukin-10; IL-1β—interleukin-1 beta; IL-6—interleukin-6; CRP—*C*-reactive protein; PGE2—prostaglandin E2; COX-2—cyclooxygenase-2; ER—endoplasmic reticulum; BiP—binding immunoglobulin protein; PERK—protein kinase R-like endoplasmic reticulum kinase; ATF6—activating transcription factor 6; NLRP3—NLR family pyrin domain containing 3; RAGE—receptor for advanced glycation end products; PI3K/AKT—phosphoinositide 3-kinase/protein kinase B pathway; TLR4—Toll-like receptor 4; Bax—Bcl-2-associated X protein; Bcl-2—B-cell lymphoma 2; PCNA—proliferating cell nuclear antigen; Nrf2—nuclear factor erythroid 2-related factor 2; Keap1—Kelch-like ECH-associated protein 1; HO-1—heme oxygenase-1; NQO1—NAD(P)H quinone dehydrogenase 1; Nfe2l2—nuclear factor erythroid 2-like 2; Hmox-1—heme oxygenase 1; Cat—catalase gene; Sod2—superoxide dismutase 2; GPx-1—glutathione peroxidase 1; StAR—steroidogenic acute regulatory protein; 3β-HSD—3β-hydroxysteroid dehydrogenase; LH—luteinizing hormone; GPER—G protein-coupled estrogen receptor; ERK—extracellular signal-regulated kinase; EIF2S1—eukaryotic translation initiation factor 2 subunit 1; TC—total cholesterol; TG—triglycerides; LDL-C—low-density lipoprotein cholesterol; HDL-C—high-density lipoprotein cholesterol; TUNEL—terminal deoxynucleotidyl transferase dUTP nick-end labeling.

## Data Availability

The original contributions presented in this study are included in the article. Further inquiries can be directed to the corresponding author.

## Abbreviations

The following abbreviations are used in this manuscript:

3β-HSD	3β-hydroxysteroid dehydrogenase
8-OHdG	8-hydroxy-2′-deoxyguanosine
AA	Ascorbic acid
AFP	Alpha-fetoprotein
ALP	Alkaline phosphatase
ALT	Alanine aminotransferase
AMPA	Aminomethylphosphonic acid
AOPP	Advanced oxidation protein products
AST	Aspartate aminotransferase
ATF6	Activating transcription factor 6
ATP	Adenosine triphosphate
Bax	Bcl-2-associated X protein
Bcl-2	B-cell lymphoma 2
BiP	Binding immunoglobulin protein
BS	Black seed/Nigella sativa
BW	Body weight
CAT	Catalase
CC BY	Creative Commons Attribution
CGA	Chlorogenic acid
CHOL	Total cholesterol
CK-MB	Creatine kinase-MB
COX-2	Cyclooxygenase-2
CoQ10	Coenzyme Q10
CRP	*C*-reactive protein
cTn-I	Cardiac troponin I
DNA	Deoxyribonucleic acid
DW	Distilled water/dry weight—w tekście przy dawkowaniu ekstraktu
EFSA	European Food Safety Authority
EIF2S1	Eukaryotic translation initiation factor 2 subunit 1
EPA	Environmental Protection Agency
ER	Endoplasmic reticulum
ERK	Extracellular signal-regulated kinase
EUG	Eugenol
GB	Ginkgo biloba
GBH/GBHs	Glyphosate-based herbicide(s)
GLY	Glyphosate
GPER	G protein-coupled estrogen receptor
GPx	Glutathione peroxidase
GR	Glutathione reductase
GSH	Glutathione
GSH-Px	Glutathione peroxidase
GSSG	Oxidized glutathione
GSSG-Red	Glutathione reductase
GST	Glutathione S-transferase
HA	Humic acid
Hb	Hemoglobin
HCT	Hematocrit
HDL	High-density lipoprotein
HDL-C	High-density lipoprotein cholesterol
HES	Hesperidin
HF	Hawthorn-leaf flavonoid
HO-1/Hmox-1	Heme oxygenase-1/heme oxygenase 1 gene
IARC	International Agency for Research on Cancer
IFN-γ	Interferon-gamma
IGF-1	Insulin-like growth factor-1
IL-1β	Interleukin-1 beta
IL-4	Interleukin-4
IL-6	Interleukin-6
IL-8	Interleukin-8
IL-10	Interleukin-10
Keap1	Kelch-like ECH-associated protein 1
KIM-1	Kidney injury molecule-1
LDL	Low-density lipoprotein
LDL-C	Low-density lipoprotein cholesterol
LH	Luteinizing hormone
LI	Licorice extract/Glycyrrhiza glabra
LPO	Lipid peroxidation
LuO	Linum usitatissimum oil
MAPK/ERK1/2	Mitogen-activated protein kinase/extracellular signal-regulated kinase 1/2
MDA	Malondialdehyde
ME	Melatonin
MO	Moringa oleifera
MT	Metallothionein
MT-I	Metallothionein I
MT-II	Metallothionein II
NAC	*N*-acetylcysteine
NAD(P)H	Nicotinamide adenine dinucleotide phosphate, reduced form
NEFA	Non-esterified fatty acids
NF-κB	Nuclear factor kappa B
Nfe2l2	Nuclear factor erythroid 2-like 2
NLRP3	NLR family pyrin domain-containing 3
NO	Nitric oxide
NOS2	Nitric oxide synthase 2
NPY	Neuropeptide Y
NQO1	NAD(P)H quinone dehydrogenase 1
Nrf2	Nuclear factor erythroid 2-related factor 2
OS	Oxidative stress
OSI	Oxidative stress index
PCNA	Proliferating cell nuclear antigen
PERK	Protein kinase R-like endoplasmic reticulum kinase
PGE2	Prostaglandin E2
PI3K/AKT	Phosphoinositide 3-kinase/protein kinase B pathway
PTH	Parathormone
QE	Quercetin
RAGE	Receptor for advanced glycation end products
RBC/RBCs	Red blood cell(s)
ROS	Reactive oxygen species
SANRA	Scale for the Assessment of Narrative Review Articles
SOD	Superoxide dismutase
StAR	Steroidogenic acute regulatory protein
SY	Selenium yeast
T3	Triiodothyronine
T4	Thyroxine
TA	Tannic acid
TAC	Total antioxidant capacity
TAS	Total antioxidant status
TC	Total cholesterol
TGF-β	Transforming growth factor beta
TG	Triglycerides
TLR4	Toll-like receptor 4
TNF-α	Tumor necrosis factor-alpha
TOS	Total oxidant status
TP	Total protein
Tre	Trehalose
TUNEL	Terminal deoxynucleotidyl transferase dUTP nick-end labeling
vitB12	Vitamin B12
vitC	Vitamin C
vitE	Vitamin E
WBC/WBCs	White blood cell(s)
XYPAE	*Xylopia aethiopica* extract
Zn	Zinc
γ-GT	Gamma-glutamyl transferase
